# Effect of Progranulin on Proliferation and Differentiation of Neural Stem/Progenitor Cells after Oxygen/Glucose Deprivation

**DOI:** 10.3390/ijms23041949

**Published:** 2022-02-09

**Authors:** Ichiro Horinokita, Hideki Hayashi, Takamasa Nagatomo, Yuna Fushiki, Yui Iwatani, Norio Takagi

**Affiliations:** Department of Applied Biochemistry, Tokyo University of Pharmacy and Life Sciences, 1432-1 Horinouchi, Hachioji-shi, Hachioji 192-0392, Japan; ihorinokita@outlook.jp (I.H.); hhayashi@toyaku.ac.jp (H.H.); taka04.n@gmail.com (T.N.); pushi0605@icloud.com (Y.F.); yiwatani@toyaku.ac.jp (Y.I.)

**Keywords:** cerebral ischemia, progranulin, neural stem/progenitor cells, Akt/GSK-3β, neurite outgrowth

## Abstract

We previously demonstrated that sivelestat, a selective neutrophil elastase inhibitor, attenuates the cleavage of progranulin (PGRN) and ischemia-induced cell injury in the brain. To obtain further insight into the role of PGRN, in the present study we evaluated the direct effects of sivelestat and recombinant PGRN (rPGRN) on the proliferation and differentiation of neural stem cells in cultures of neural stem/progenitor cells (NS/PC) under the ischemic condition in vitro. We demonstrated that oxygen/glucose deprivation (OGD)-induced cell proliferation of NS/PC was increased by rPGRN treatment. In addition, this increase was accompanied by increased phosphorylation of Akt and GSK-3β (Ser9) after OGD. But none of these responses occurred by treatment with sivelestat. Therefore, activation of the Akt/GSK-3β pathway could well be involved in this proliferative effect of rPGRN. Although OGD and reoxygenation-induced changes in the differentiation of NS/PC into neurons or astrocytes was not affected by treatment with rPGRN or sivelestat, it is noteworthy that rPGRN enhanced neurite outgrowth of β3-tubulin-positive neurons that had differentiated from the NS/PC. These findings suggest that enhancement of proliferation of endogenous NS/PC and neurite outgrowth of differentiated neurons from NS/PC by PGRN could be useful for a new therapeutic approach for cerebral ischemia.

## 1. Introduction

Neural stem cells (NSCs) differentiate into neurons, astrocytes, and oligodendrocytes via neural progenitor cells (NPCs). Cell proliferation and differentiation of neural stem/progenitor cells (NS/PC) in the subventricular zone and hippocampal dentate gyrus in the adult brain are consistently activated [1,2]. Neurogenesis induced by cerebral ischemia increases transiently seven days after microsphere embolism, as shown in our previous study [3]. Neurons are considered to migrate to the injury site and integrate into neural circuits [4]. However, as described above, neurogenesis after cerebral ischemia is transient and does not reach a sufficient amount to recover function [5,6]. In addition, only a small percentage of newborn neurons can survive due to the inflammatory response that occurs after cerebral ischemia. Therefore, continuous induction of newly generated endogenous neurons will be needed to improve ischemic injury.

Progranulin (PGRN), which is cleaved to granulin by neutrophil elastase, is crucial in diverse cellular functions, such as cell proliferation and embryonic development, regulation of inflammatory response, and protection against vascular and neuronal disorders [7,8,9,10]. It has been reported that PGRN contributes to cell proliferation through extracellular signal-regulated kinase (ERK), phosphoinositide 3-kinase (PI3-K), and focal adhesion kinase (FAK) pathways in cancer cells [11]. Based on these findings and the results in our previous study, PGRN may attenuate the decrease in the number of NS/PC early after cerebral ischemia and thus contribute to protection against ischemic injury.

In the present study, we examined the effects of sivelestat, a selective neutrophil elastase inhibitor, on post-stroke neurogenesis in vivo in the ischemic brain, because PGRN is cleaved to granulin by neutrophil elastase. Then we examined the direct effects of sivelestat and rPGRN on the proliferative and differentiation potential of cultured NS/PC under the OGD condition.

## 2. Results

### 2.1. Effects of Sivelestat on the Number of Doublecortin (DCX)-, Ki67-, and NeuroD-Positive Cells after Cerebral Ischemia

**Figure 1:**
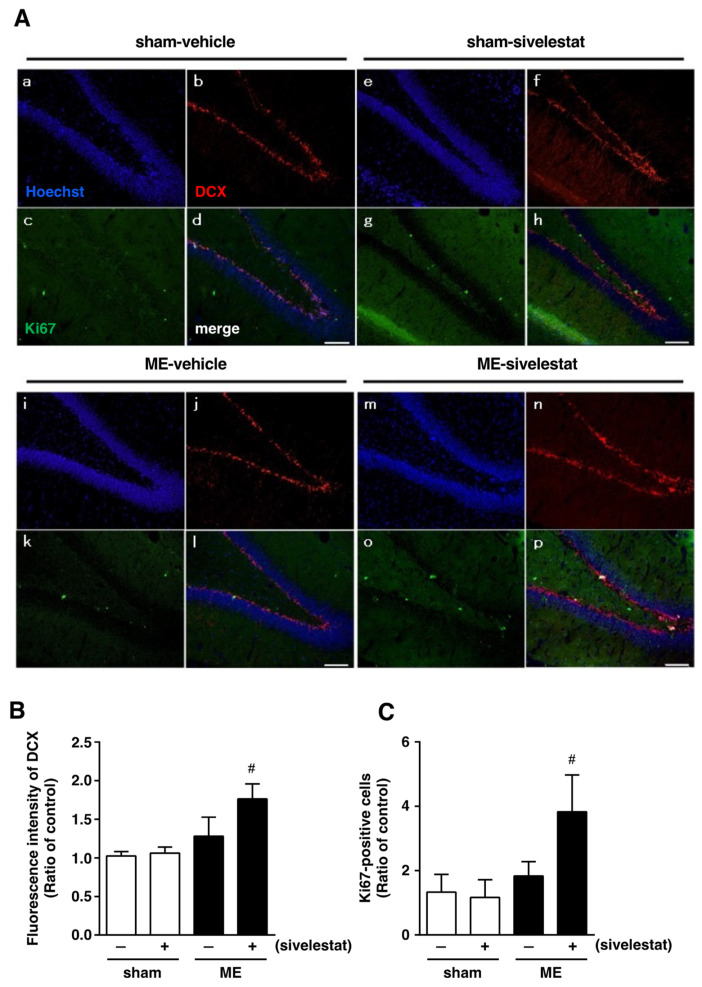
(**A**) Images of triple staining (merge, (**d**,**h**,**l**,**p**)) for DCX (red, (**b**,**f**,**j**,**n**)) and Ki67 (green, (**c**,**g**,**k**,**o**)), and with Hoechst 33342 (blue, (**a**,**e**,**I**,**m**)) for the vehicle-treated sham and ME groups and sivelestat-treated sham and ME groups on day one after surgery are shown. The scale bar represents 100 μm. (**B**) The fluorescence intensity of DCX in the vehicle-treated (−) sham- (white bars) and ME-operated (black bars) groups and sivelestat-treated (+) sham- (white bars) and ME-operated (black bars) groups on day one after surgery was measured. Five sections were made per animal, and the average of five sections per animal was calculated. The values for fluorescence intensity of DCX cells are presented as the mean ± SD (*n* = 5 each). (**C**) The number of Ki67-positive cells in the hippocampal dentate gyrus of the vehicle-treated (−) sham- (white bars) and ME-operated (black bars) groups and sivelestat-treated (+) sham- (white bars) and ME-operated (black bars) groups on day one after surgery was counted. The results are expressed as the mean ratio of the non-operated (control) group ± SD (*n* = 5 each). # Significant difference from the vehicle-treated ME group (*p* < 0.05).

**Figure 2:**
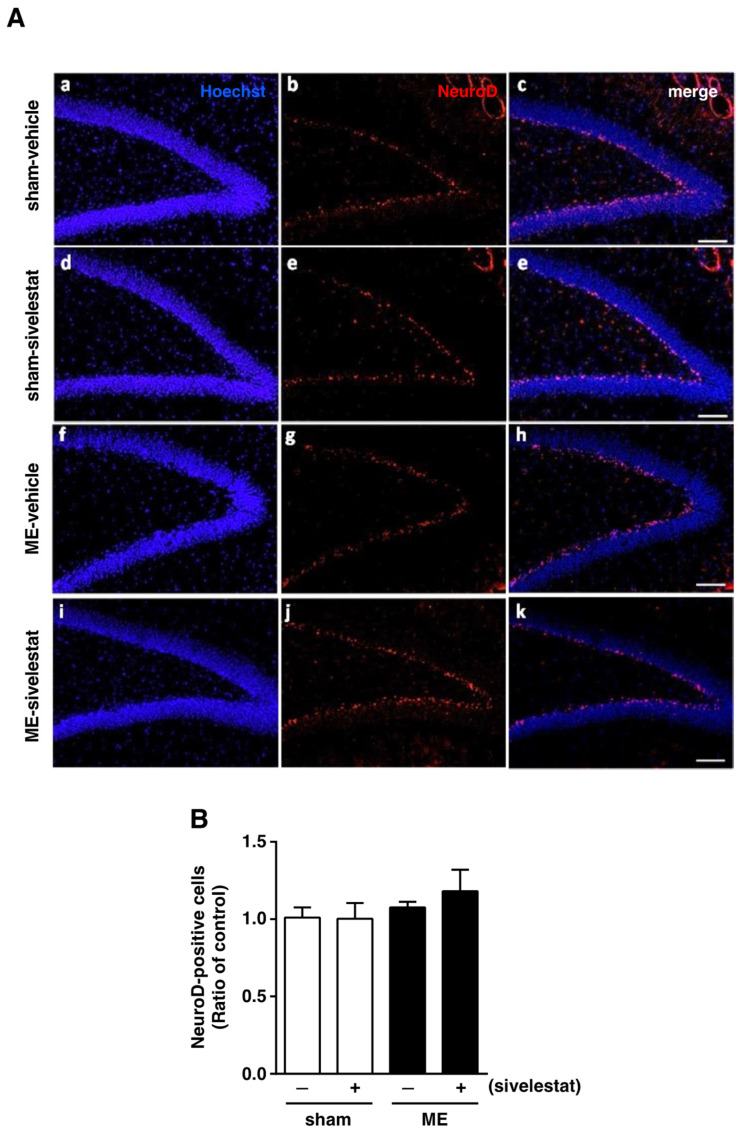
(**A**) Images of double-stained sections (merge, (**c**,**e**,**h**,**k**)) for Neuro D (red, (**b**,**e**,**g**,**j**)), and with Hoechst 33342 (blue, (**a**,**d**,**f**,**i**)) for the vehicle-treated sham and ME groups and sivelestat-treated sham and ME groups on day one after surgery are shown. The scale bar represents 100 μm. (**B**) The number of NeuroD-positive cells in the hippocampal dentate gyrus of the vehicle-treated (−) sham- (white bars) and ME-operated (black bars) groups and sivelestat-treated (+) sham- (white bars) and ME-operated (black bars) groups on day one after surgery was counted. Results are expressed as the mean ratio of the non-operated (control) group ± SD (*n* = 5 each).

We first examined the effect of sivelestat on the fluorescence intensity of DCX-positive cells and on the number of Ki67-positive cells in the hippocampal dentate gyrus on day one after microsphere-induced cerebral embolism (ME). DCX and Ki67 were used as markers for neural progenitor cells and proliferating cells, respectively. The DCX fluorescence intensity and the number of Ki67-positive cells in the sham-operated group were not affected by the administration of sivelestat (Figure 1). Although neither the former nor latter was increased in the ME-vehicle group compared with the sham-vehicle group, the treatment with sivelestat significantly increased both after ME (Figure 1). We further examined the effects of sivelestat on the number of NeuroD-positive cells in the hippocampal dentate gyrus on day one after ME. NeuroD was used as a marker for neuronal differentiation-related proteins. The number of NeuroD-positive cells did not differ between sham- and ME-operated groups with or without sivelestat treatment (Figure 2).

### 2.2. Effects of Sivelestat and rPGRN on Proliferation of Neural Stem/Progenitor Cells (NS/PC) under the Oxygen and Glucose Deprivation (Ogd) Condition

**Figure 3:**
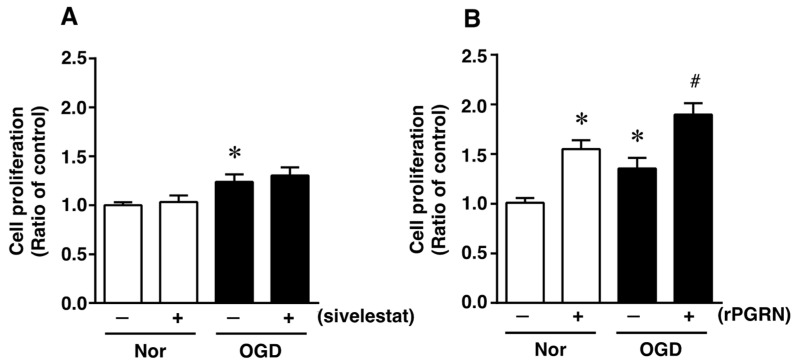
Cell proliferation of neural stem/progenitor cells in the vehicle-treated (−) normoxia (white bars) and OGD (black bars) groups and sivelestat (**A**)- or rPGRN (**B**)-treated (+) normoxia (white bars) and OGD (black bars) groups is shown. Cell proliferation of neural stem/progenitor cells was determined by performing the XTT assay. Results are expressed as the mean ratio of the non-treated (control) group ± SD (*n* = 5 each). * Significant difference from the vehicle-treated normoxic group (*p* < 0.05). # Significant difference from the vehicle-treated OGD group (*p* < 0.05).

To investigate what might possibly be the mechanism of action of the elastase inhibitor in vivo, we next focused on the direct effects of sivelestat and PGRN on NS/PC under the in vitro ischemic condition. At first, the effect of sivelestat on the proliferation of NS/PC after 24 h of OGD was determined by performing the XTT assay. Cell proliferation was increased under the OGD condition compared with that under normoxia, but this increased cell proliferation was not affected by treatment with sivelestat (Figure 3A). We further examined the effect of recombinant progranulin (rPGRN) on the proliferation of NS/PC after 24 h of OGD. Cell proliferation was significantly increased under the normoxic condition by the treatment with rPGRN (Figure 3B). The increase in cell proliferation under the OGD condition was further enhanced by treatment with rPGRN (Figure 3B).

### 2.3. Effects of Sivelestat and rPGRN on Akt/GSK-3β Pathway of NS/PC after OGD

**Figure 4:**
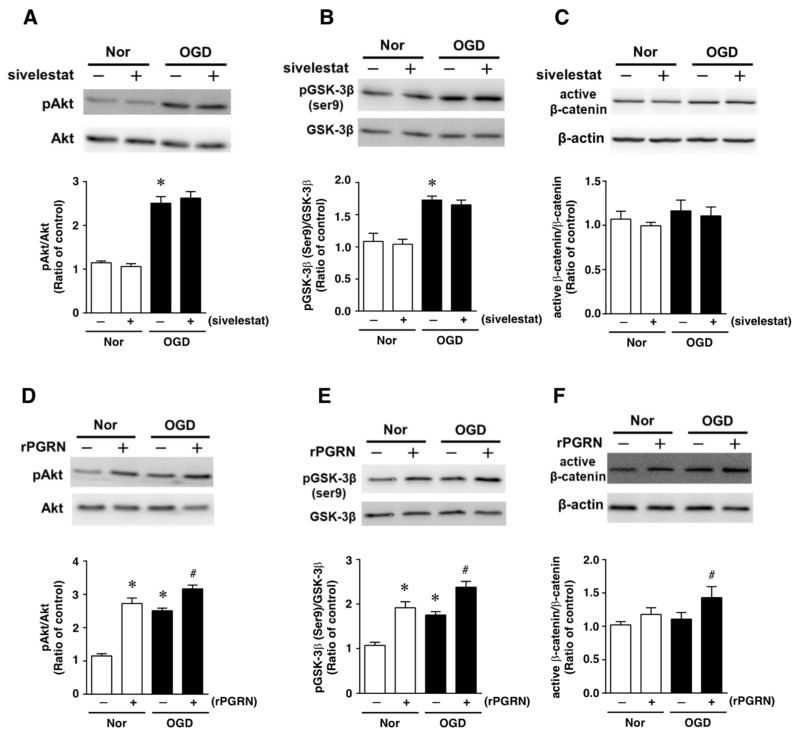
Levels of pAkt (**A**,**D**), pGSK-3β (**B**,**E**), and active-β-catenin (**C**,**F**) in the vehicle-treated (−) normoxia (white bars) and OGD (black bars) groups and sivelestat- (**A**–**C**) or rPGRN (**D**–**F**)-treated (+) normoxia (white bars) and OGD (black bars) groups at 24 h after OGD are shown. Bands corresponding to pAkt, pGSK-3β, and active-β-catenin were scanned, and the scanned bands were normalized by total Akt, GSK-3β, and β-actin on the same blot, respectively. Results are expressed as the mean ratio of the non-treated (control) group ± SD (*n* = 5 each). * Significant difference from the vehicle-treated normoxic group (*p* < 0.05). # Significant difference from the vehicle-treated OGD group (*p* < 0.05).

Next, the effects of sivelestat on the phosphorylation of Akt and GSK-3β (Ser9) were examined under the OGD condition. The phosphorylation of either one was increased under the OGD condition compared with that under normoxia. These increases in phosphorylation were not affected by treatment with sivelestat (Figure 4A,B). Furthermore, the level of active β-catenin was not different between normoxia and the OGD condition regardless of treatment or not with sivelestat (Figure 4C).

We next examined the effects of rPGRN on the phosphorylation of Akt and GSK-3β (Ser9), and the level of active β-catenin. The phosphorylation levels of Akt and GSK-3β (Ser9) were increased under the normoxic condition by treatment with rPGRN (Figure 4D,E). Although the phosphorylation of either was also increased by OGD without rPGRN treatment, this increase under the OGD condition was further enhanced by the treatment with rPGRN (Figure 4D,E). The level of active β-catenin was not altered under the OGD condition compared with that under the normoxic condition; however, treatment with rPGRN increased the level of active β-catenin under the OGD condition (Figure 4F).

### 2.4. Effects of Sivelestat and rPGRN on the Expression of GFAP and NeuroD1 mRNAs, as Cell Differentiation Markers, after OGD/R

**Figure 5:**
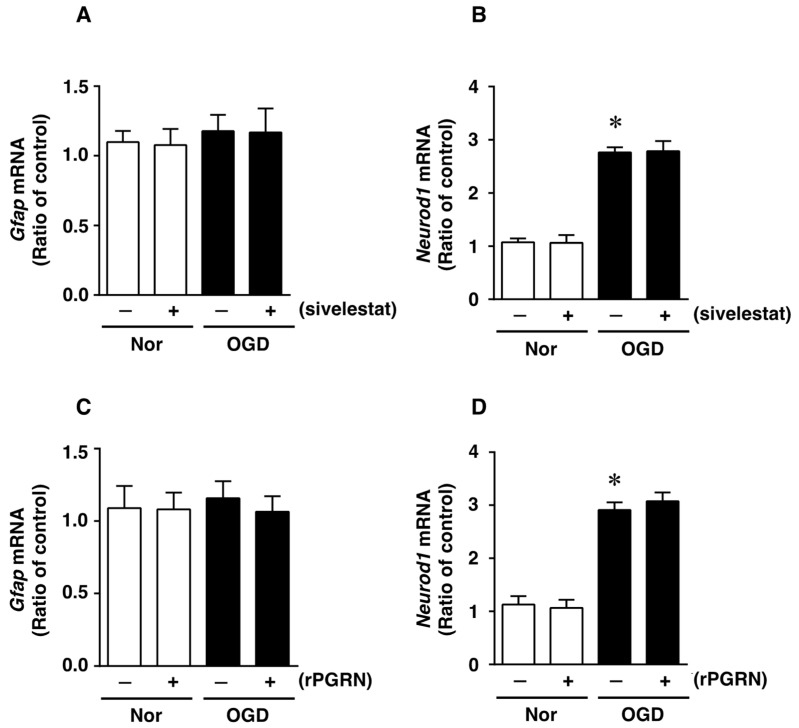
Levels of Gfap (**A**,**C**) and NeuroD1 (**B**,**D**) mRNAs in the vehicle-treated (−) normoxia (white bars) and OGD/R (black bars) groups and sivelestat- (**A**,**B**) or rPGRN (**C**,**D**)-treated (+) normoxia (white bars) and OGD/R (black bars) groups at 72 h after OGD/R are shown. Results are expressed as the mean ratio of the normoxia or OGD/R to the control group ± SD (*n* = 5 independent experiments). * Significant difference from the vehicle-treated normoxic group (*p* < 0.05).

We next investigated whether treatment with sivelestat or rPGRN would affect the expression of glial fibrillary acidic protein (GFAP) mRNA, as an astrocytic marker of cell differentiation, after OGD/R. The expression of GFAP mRNA was not altered under the OGD/R condition compared with that under the normoxic condition (Figure 5A). In addition, there was no change in the expression of GFAP mRNA regardless of treatment or not with sivelestat (Figure 5A). In contrast, the expression of NeuroD1 mRNA was enhanced by the OGD/R condition during cell differentiation, and this increased expression was not affected by treatment with sivelestat (Figure 5B). Furthermore, the expression of GFAP mRNA was not affected under normoxia regardless of treatment or not with rPGRN (Figure 5C). The enhanced expression of NeuroD1 mRNA under OGD/R was not affected by treatment with rPGRN (Figure 5D).

### 2.5. Effects of Sivelestat and rPGRN on Differentiation of NS/PC after OGD/R

**Figure 6:**
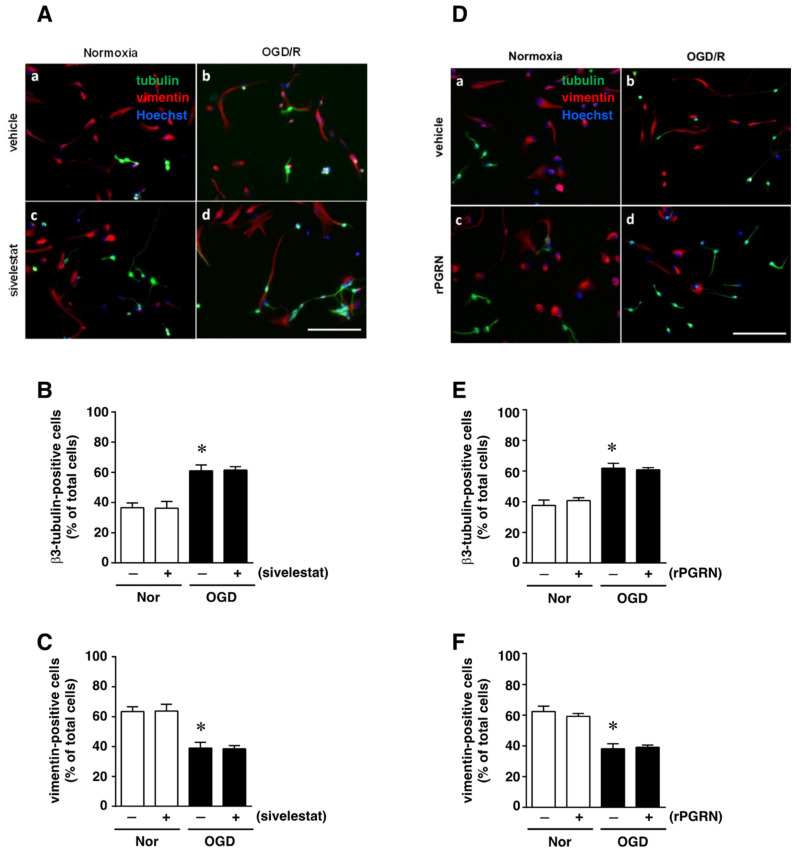
(**A**,**D**) Images of double staining for vimentin (red) and β3-tubulin (green), and with Hoechst3342 (blue), for the normoxia-vehicle (**A**-**a**,**D**-**a)**, normoxia-sivelestat (**A**-**c**), and -rPGRN (**D**-**c**) groups, as well as those for the OGD/R-vehicle (**A**-**b**,**D**-**b**), OGD/R-sivelestat (**A**-**d**), and OGD/R-rPGRN (**D**-**d**) groups are shown. The scale bar represents 100 μm. The numbers of β3-tubulin- (**B**,**E**) and vimentin-positive cells (**C**,**F**) for vehicle-treated (−) normoxia (white bars) and OGD/R (black bars) groups and for sivelestat (**B**,**C**)- or rPGRN (**E**,**F**)-treated (+) normoxia (white bars) and OGD/R (black bars) groups at 96 h after OGD/R were counted. The results are expressed as the percentage of vimentin-positive cells among the total number of Hoechst-positive cells and that of β3-tubulin-positive cells among the total number of Hoechst-positive cells, and as the means ± SD (*n* = 5 independent experiments. The total number of Hoechst-positive cells counted was 7148 (**A**-**a**; vehicle) and 7464 (**D**-**a**; vehicle); 7046 (**A**-**c**; sivelestat) and 7463 (**D**-**c**; rPGRN) for the normoxia group; 7614 (**A**-**b**; vehicle) and 7658 (**D**-**b**; vehicle); and 7600 (**A**-**d**; sivelestat) and 7590 (**D**-**d**; rPGRN) for the OGD/R group. * Significant difference from the vehicle-treated normoxic group (*p* < 0.05).

We next examined the effect of sivelestat on the ability of NS/PC to differentiate into neurons as β3-tubulin-positive cells or into astrocytes as vimentin-positive cells after OGD/R. The ability for differentiation into neurons under OGD/R was enhanced compared with that of the normoxia-vehicle-treated group (Figure 6A,B). This increase was not affected by treatment with sivelestat (Figure 6A,B). On the other hand, the ability to differentiate into astrocytes under the OGD/R condition was reduced compared with that of the normoxia-vehicle-treated group, and this decrease was not affected by treatment with sivelestat under the OGD/R condition (Figure 6A,C). In addition, the changes in differentiation into neurons and astrocytes under the OGD/R condition were not affected by treatment with rPGRN (Figure 6D–F).

**Figure 7:**
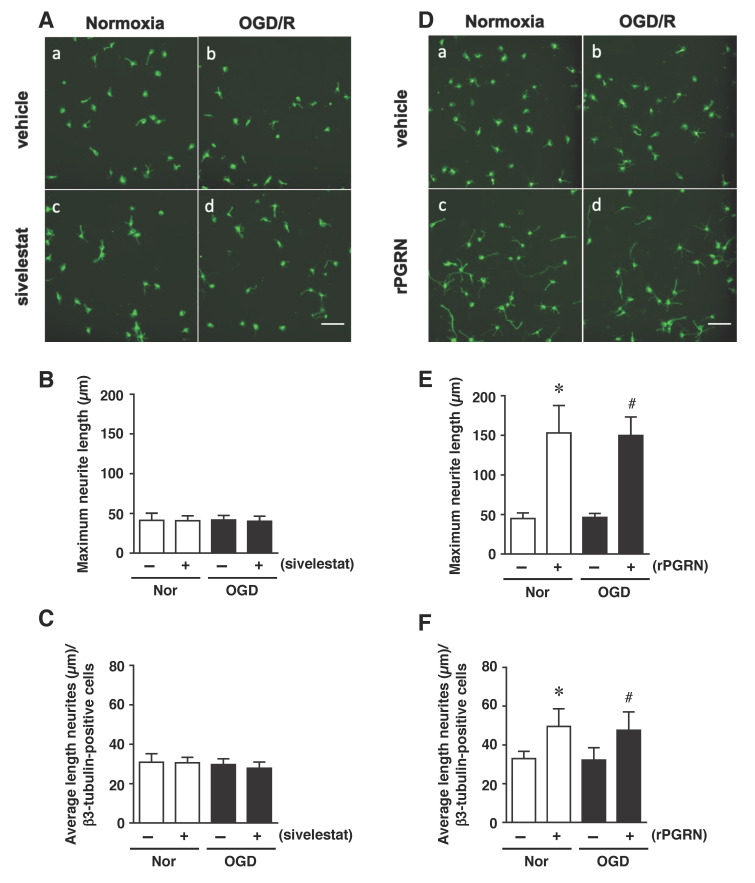
(**A**,**D**) Staining results for β3-tubulin in the normoxia-vehicle group (**A**-**a**,**D**-**a**), normoxia-sivelestat (**A**-**c**), and -rPGRN (**D**-**c**) groups, as well as those for OGD/R-vehicle (**A**-**b**,**D**-**b**), OGD/R-sivelestat (**A**-**d**), and OGD/R-rPGRN (**D**-**d**) groups are shown. The scale bar represents 100 μm. The maximum neurite length (**B**,**E**) and average length of neurites (**C**,**F**) for the vehicle-treated (−) normoxia (white bars) and OGD/R (black bars) groups and sivelestat (**B**,**C**) or rPGRN (**E**,**F**)-treated (+) normoxia (white bars) and OGD/R (black bars) groups at 96 h after OGD/R were measured. The results are expressed as the means (μm) ± SD of these cells among the total number of β3-tubulin-positive cells (*n* = 5 independent experiments. The total number of β3-tubulin-positive cells counted was 2738 (**A**-**a**; vehicle) and 2692 (**D**-**a**; vehicle); 2709 (**A**-**c**; sivelestat), and 2896 (**D**-**c**; rPGRN) for the normoxia group; 4654 (**A**-**b**; vehicle) and 4702 (**D**-**b**; vehicle); and 4665 (**A**-**d**; sivelestat) and 4621 (**D**-**d**; rPGRN) for the OGD/R group. * Significant difference from the vehicle-treated normoxic group (*p* < 0.05). # Significant difference from the vehicle-treated OGD group (*p* < 0.05).

Finally, we examined the effects of sivelestat and rPGRN treatment on neurite length of β3-tubulin-positive cells under the OGD/R condition. The results demonstrated that the neurite length was not changed under the OGD/R condition regardless of treatment or not with sivelestat compared to the others (Figure 7A–C). In contrast, neurite growth of differentiated NS/PC was similarly enhanced by rPGRN treatment under both normoxia and the OGD/R condition compared with that for the vehicle-treated groups (Figure 7D–F).

## 3. Discussion

Our previous study demonstrated that treatment with an elastase inhibitor, sivelestat, inhibited PGRN cleavage and could suppress the progression of ischemic brain injury [12]. To further investigate therapeutic strategies for ischemic stroke, we focused on the roles of PGRN in proliferation and differentiation of NS/PC after cerebral ischemia. We first examined whether sivelestat would affect the proliferation and differentiation potential of neural stem cells in the dentate gyrus of the hippocampus. The administration of sivelestat significantly increased the fluorescence intensity of DCX and the number of Ki67-positive cells in the hippocampal dentate gyrus, whereas the number of NeuroD-positive cells did not change. These findings suggest that the differentiation into neurons was not enhanced by sivelestat after cerebral ischemia. Therefore, we next investigated the direct effect of treatment with sivelestat or rPGRN on the proliferation of isolated NS/PC cultured under the OGD condition. Although proliferation of isolated NS/PC was slightly increased under OGD, this proliferation was not enhanced by treatment with sivelestat. Interestingly, the increase in proliferation of NS/PC induced by OGD was further enhanced by rPGRN treatment. It was reported that neural stem cells derived from PGRN KO mice have reduced ability to proliferate compared with those of wild-type mice and that the addition of exogenous PGRN enhances cell proliferation [13]. These findings suggest that the enhancement of cell proliferation of ME rats administered sivelestat could have been caused by sivelestat-mediated suppression of PGRN cleavage, resulting in an increased level of PGRN.

Neurogenesis occurring after cerebral ischemia is regulated by various intracellular signaling pathways. We and others demonstrated that PI3-K/Akt/GSK-3β signaling in neural stem cells is involved in neurogenesis in response to cerebral ischemia [14,15]. Furthermore, it has been reported that PGRN contributes to cell proliferation by acting through the ERK, PI3-K, and FAK pathways in cancer cells [16]. Therefore, we investigated whether sivelestat or rPGRN treatment would affect the proliferation of NS/PCs via the Akt/GSK-3β signaling pathway under the in vitro ischemic condition. Treatment with rPGRN, but not with sivelestat, increased the phosphorylation of Akt and GSK-3β (ser9) compared with that of the untreated group. In this sense, neurogenesis via phosphorylated GSK-3β, which is the inactive form of GSK-3β, after PGRN treatment is also observed in human and mouse neural stem cells [13,16]. These findings may also rely on a mechanism similar to that observed in the present study. Furthermore, we also demonstrated that rPGRN increased the level of active (non-phosphorylated) β-catenin downstream of GSK-3β under the OGD condition. Therefore, our findings suggest that the enhanced proliferation of NS/PCs by sivelestat treatment after cerebral ischemia was possibly due to PGRN, which escapes elastase-induced cleavage. In addition, PGRN activates the Akt/GSK-3β signaling pathway in neural stem cells.

We further demonstrated that the enhanced differentiation of NS/PC into neurons under the OGD condition was not affected by treatment with sivelestat or rPGRN. The ability of differentiation into astrocytes was also not affected, regardless of treatment. In this sense, exogenous PGRN did not affect asymmetric division, which differentiates into neurons, astrocytes, and oligodendrocytes in neural stem cells derived from PGRN-deficient mice [13]. It has been also reported that self-repairing by neurons, which are formed from stem cells, does not occur in brain injury or neurodegenerative diseases [17]. During spinal cord injury, although endogenous neural stem cells proliferate in response to the injury, they all differentiate into astrocytes instead of differentiating into neurons [18]. These findings imply the involvement of a microenvironment in the brain that inhibits neurogenesis. As it is important to not only promote neurogenesis itself but also promote the survival and maintenance of newly generated neurons, we next focused on the role of PGRN in the generation of new neurons from NS/PC under the in vitro ischemic condition.

It is noteworthy that treatment with rPGRN enhanced neurite outgrowth of NS/PC-derived neurons, although the ability of NS/PC to differentiate was not affected by treatment with rPGRN. In this sense, neurite outgrowth of neurons in primary culture, derived from PGRN-deficient mice, was less than that of those from WT mice [19]. In addition, treatment with PGRN has been shown to enhance neurite outgrowth of neurons in primary culture [20]. Both groups suggested that GSK-3β-mediated signaling might contribute to PGRN-induced neurite outgrowth [19,20]. Others showed that treatment of dorsal root ganglion cells with a GSK-3β inhibitor reduced neurite outgrowth [21]. Therefore, our findings suggest that the enhancement of neurite outgrowth by rPGRN may have been mediated by phosphorylation of GSK-3β (Ser9), an inactive form of GSK-3β. The active form of GSK-3β phosphorylates collapsin response mediator protein-2 (CRMP2) and inactivates it. Non-phosphorylated CRMP-2, which is an active form, is reported to be involved in neurite outgrowth by binding to tubulin and promoting microtubule polymerization [22]. Furthermore, inhibition of GSK-3β by BDNF leads to a decrease in the phosphorylation of CRMP2, thus promoting axonal outgrowth [23]. Although it will be necessary to investigate the role of CRMP2 under the pathologic conditions, CRMP2, acting downstream of GSK-3β, may be involved in the mechanisms of PGRN-mediated neurite outgrowth of differentiated NS/PC. In central nervous system diseases such as dementia and Alzheimer’s disease, neurite loss is observed, and so it has been thought that induction of neurite outgrowth would be useful. The findings obtained from the present study suggest that PGRN plays a pivotal role in the ability of NS/PC to proliferate and in neurite growth in the ischemic condition and, therefore, may be potentially used as a new therapeutic strategy for certain nervous system disorders.

**Figure 8:**
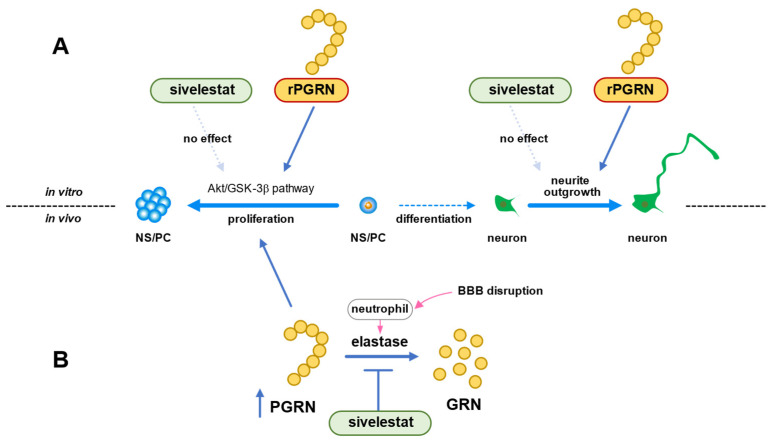
(**A**) Schematic diagram of effects of PGRN or sivelestat on proliferation of NS/PC and neurite growth of neurons that had differentiated from NS/PC in vitro ischemic condition. (**B**) Schematic diagram of effects of sivelestat on proliferation of NS/PC after in vivo cerebral ischemia.

In conclusion, we demonstrated that the Akt/GSK-3β signaling pathway was involved in the mechanism of NS/PC proliferation induced by PGRN under the in vitro ischemic condition (Figure 8A). The ability of NS/PC to differentiate was not affected by PGRN. However, this cysteine-rich glycoprotein enhanced neurite growth of neurons that had differentiated from NS/PC even under the OGD/R condition (Figure 8A). Therefore, our data suggested that the increase in the number of proliferating cells by treatment with sivelestat after cerebral ischemia was not due to the direct effect of sivelestat on NS/PC but possibly due to the indirect effect of PGRN, whose expression was increased by treatment with the elastase inhibitor sivelestat (Figure 8B). Our findings thus suggest that enhancement of proliferation of endogenous NS/PC and neurite outgrowth of differentiated neurons from NS/PC by PGRN might be useful as a new therapeutic strategy for cerebral ischemia.

## 4. Materials and Methods

### 4.1. Materials

Elastin-Congo Red (Cat. No. E164) was purchased from Elastin Product (Owensville, MO, USA). Siverestat was obtained from Nipro (Osaka, Japan).

### 4.2. Model of Microsphere-Induced Cerebral Embolism in Rats

In the present study male Wistar rats weighing between 220 and 250 g (Charles River Japan Inc., Tsukuba, Japan) were used for a cerebral ischemic model; and Wistar rats (Japan SLC, Shizuoka, Japan) at embryonic day 14, for preparing cell cultures. The rats were maintained under controlled conditions at 23 ± 1 °C and 55 ± 5% humidity with a light cycle of 12-h light/12-h darkness, had free access to food and water, and were maintained according to the National Institute of Health Guide for the Care and Use of Laboratory Animals and the Guidance for Experimental Animal Care issued by the Prime Minister’s Office of Japan, Tokyo, Japan. The study was approved by the Committee of Animal Care and Welfare of Tokyo University of Pharmacy and Life Sciences, Tokyo, Japan.

Microsphere-induced cerebral embolism (ME) was performed by the method described previously [24,25]. Anesthesia was induced with 5% isoflurane and maintained with 2.5% isoflurane. The right external carotid and pterygopalatine arteries were temporarily occluded with strings. Immediately thereafter, a needle connected to a polyethylene catheter (TORAY Feeding Tube, Chiba, Japan) was inserted into the right common carotid artery, and then 700 microspheres (45.0 μm in diameter; Polysciences Inc., Warrington, PA, USA), suspended in 20% dextran solution (150 µL), were injected into the right internal carotid artery through the cannula. After the injection, the needle was removed, and the puncture wound was then repaired with surgical glue. The rats that underwent a sham operation received the same volume of vehicle without microspheres. Non-operated rats were used as naïve control rats in the present study.

On day 1 after the surgery, neurological deficits of the operated rats were scored on the basis of paucity of movement, truncal curvature, and forced circling during locomotion according to the criteria described previously [24,25]. The score of each neurological deficit was rated from 3 to 0 (3, very severe; 2, severe; 1, moderate; 0, little or none). The rats with a total score of 7–9 points on day 1 after cerebral embolism were used in this study.

### 4.3. Drug Administration

Sivelestat, which is a selective inhibitor of neutrophil elastase, was dissolved in phosphate-buffered saline (PBS) and administered (50 mg/kg) intravenously twice, once just after the surgery for ME and then subcutaneously 8 h after ME. The dose of sivelestat and this type of drug administration were based on the reports of Ikegama et al. [26] and Tonai et al. [27], and on results of our previous study [12].

### 4.4. Histochemical Analysis

On day 1 after surgery, ME- and sham-operated rats with or without sivelestat treatment were perfused via the heart with 4% paraformaldehyde in 0.1 mol/L phosphate buffer. The brains were quickly removed and immersed in 30% sucrose in 0.1 mol/L phosphate buffer and then cut into 5-mm-thick coronal slabs, which were subsequently embedded in Neg50 (Richard-Allan Scientific, Kalamazoo, MI, USA) and cut into 10 µm sections by using a cryostat. For immunostaining, mouse anti-Ki67 (556003, BD pharmingen, Franklin Lakes, NJ, USA), goat anti-DCX (SC-8066, Santa Cruz Biotechnology Inc., Santa Cruz, CA, USA), and goat anti-NeuroD (SC-1084, Santa Cruz Biotechnology Inc.) antibodies were used, along with AlexaFluor 488-labeled donkey anti-mouse IgG (Molecular Probes Inc., Eugene, OR, USA) and AlexaFluor 594-labeled donkey anti-goat IgG (Molecular Probes Inc.) antibodies as the secondary antibodies. Fluorescence was detected by using an Olympus fluorescence microscope (IX-71; Olympus, Tokyo, Japan). Omission of primary antibodies served as a negative control. No immunostaining was detected in this group. Fluorescent images were loaded into the MetaMorph software program (Molecular Devices, Downingtown, PA, USA). Based on background fluorescence and the size of their nucleus, antibody-labeled cells of the cerebral cortex were observed by use of the MetaMorph software program (5 sections per animal), whose areas corresponded to coronal coordinates of 8.06 to 9.70 from the interaural point.

### 4.5. Isolation of NS/PC

NPCs were prepared from rats at embryonic day 14 according to the method described previously [7,14]. Isolated cells were seeded at a density of 2 × 106 cells per non-treated 75-cm^2^ flask (Cat No. 156800 Nunc, Thermo Fisher Scientific, Waltham, MA, USA) and at a density of 5 × 10^5^ cells per non-treated 25-cm^2^ flask (Cat No. 169900 Nunc). The culture medium contained N2-max supplement (R&D Systems, Inc., Minneapolis, MN, USA), 20 ng/mL epidermal growth factor (EGF; PeproTech, Rocky Hill, NJ, USA), and 20 ng/mL basic fibroblast growth factor (bFGF; PeproTech, Rocky Hill, NJ, USA) in DMEM/F12 (Invitrogen, Waltham, MA, USA). NPCs were cultured as floating neurospheres, and the medium was exchanged for fresh medium at 4 days after culture preparation.

### 4.6. Oxygen and Glucose Deprivation (OGD) and OGD and Reoxygenation (OGD/R)

Neurospheres were cultured for 7 days and dissociated into single cells by using Accutase (Invitrogen Co., Carlsbad, CA, USA). For cultures under the OGD condition, the culture medium was replaced with DMEM without D-glucose and sodium pyruvate (Cat No. 11966025, Thermo Fisher Scientific, Waltham, MA, USA), but containing 0.5 mM L-glutamine, 20.1 mM NaHCO_3_, and N2-max supplement. Then the cultures were placed in a hypoxic chamber, which was initially flushed with a mixture of 95% N_2_ and 5% CO_2_, within a humidified modular incubator at 37 °C. The oxygen concentration in the chamber was maintained at 5% with a residual gas mixture of 5% CO_2_ and balanced nitrogen for 24 h at 37 °C. For cultures in the normoxic environment, cells as a matched control group were cultured at 37 °C in 95% atmospheric air and 5% CO_2_ for the same times as cells under the hypoxic condition. NPCs were induced to differentiate by culturing them in DMEM/F12 containing 2% B27 (Invitrogen) and 0.5% FBS. For cultures of differentiated cells under OGD with reoxygenation, the culture medium was replaced with DMEM (no D-glucose, no sodium pyruvate) containing B27 and FBS under OGD. After OGD exposure, the medium was replaced with DMEM/F12 containing 2% B27 and 0.5% FBS and cultured under the normoxic condition at 37 °C (OGD/R). Cells were treated simultaneously with sivelestat (100 µM; Nipro, Osaka, Japan), an inhibitor of elastase, or rPGRN (100 ng/mL; AG-40A-0196Y-C010, Adipogen Life Sciences, San Diego, CA, USA) and OGD exposure.

### 4.7. Cell Proliferation

Cell proliferation was assessed by performing the sodium 3′-[1-[(phenylamino)-carbony]-3,4-tetrazolium]-bis(4-methoxy-6-nitro)benzene-sulfonic acid hydrate (XTT) assay (Sigma-Aldrich, St. Louis, MO, USA), which is widely used to measure cell proliferation by detecting the redox potential of cells [28]. XTT-PMS solution (125 µL; 1 mg/mL XTT and 1.54 mg/mL PMS) was added to medium containing neural stem/progenitor cells. After 1 h of incubation at 37 °C, the absorbance was measured with a microplate reader at 450 nm. The relative cell proliferation was expressed as the ratio of the absorbance of each group against the normoxia group.

### 4.8. Western Immunoblotting

On day 1 after surgery, ME- and sham-operated rats were sacrificed by decapitation. The right hemisphere was homogenized in ice-cold buffer containing 10% sucrose, 1 mM ethylenediaminetetraacetic acid (EDTA), and protease inhibitor cocktail (Roche Diagnostics GmbH, Mannheim, Germany) in 20 mM Tris-HCl (pH 7.4). Samples were heated at 95 °C for 5 min in 10% glycerol and 2% sodium dodecyl sulfate (SDS) in 62.5 mM Tris-HCl (pH 6.8). Cultured cells were harvested in sample buffer comprising 62.5 mM Tris-HCl (pH 6.8), 10% glycerol, 2% SDS, and 5% β-mercaptoethanol and heated for 5 min at 95 °C. Western blotting was performed according to standard protocols.

The following primary antibodies from Cell Signaling Technology were used: rabbit anti-pGSK-3β (Ser9; Cat. No. 5558S), rabbit anti-GSK-3β (9315S), rabbit anti-pAkt (4060S), rabbit anti-Akt (4691S), rabbit anti-active-β-catenin (8814S), and rabbit anti-β-catenin (8480S). Also used was mouse anti-β-actin (a1978, Sigma-Aldrich Corp., St. Louis, MO, USA). Quantification was performed by using computerized densitometry (Luminograph II, ATTO Co., Tokyo, Japan) and an image analyzer (CS Analyzer, ATTO Co., Tokyo, Japan).

### 4.9. qRT-PCR

Total RNAs were extracted from cultured cells by using an RNA extraction kit, Isogen II (Nippon Gene, Tokyo, Japan) and quantified with a BioSpec-nano (Shimazu Corp., Kyoto, Japan). cDNAs were synthesized from 500 ng of total RNAs by use of ReverTra Ace^®^ qPCR RT Master Mix with gDNA Remover (Toyobo Co., Ltd., Tokyo, Japan). qRT-PCR was performed with THUNDERBIRD^®^ SYBR qPCR Mix (Toyobo Co., Ltd.) on a CFX Connect Real-Time PCR Detection System (Bio-Rad Laboratories, Hercules, CA, USA). Data were normalized to 18S rRNA mRNA expression and analyzed by the 2−ΔΔCt method. Primers used in the present study were as follows: 18 S rRNA—forward, 5′-CGGACAGGATTGACAGATTG-3′; reverse, 5′-CAAATCGCTCCACCAACTAA-3′. Neurod1—forward, 5′-GAACACGAGGCAGACAAGAA-3′; reverse, 5′-TCATCTTCATCCTCCTCCTCTC-3′. Gfap—forward, 5′-CCAGATCCGAGAACCAGCC-3′; reverse, 5′-CCGCATCTCCACCGTCTTTA-3′.

### 4.10. Immunocytochemistry

Cultured cells were fixed with 4% paraformaldehyde and blocked with 10% donkey serum and 1% bovine serum albumin in Triton X-100 in PBS. The primary antibodies used were rabbit anti-β3-tubulin (5568S, Cell Signaling Technology, Danvers, MA, USA) and mouse anti-vimentin (5741S, Cell Signaling Technology); and the secondary ones, Alexa Fluor 488-labeled goat anti-rabbit IgG (A11034; Invitrogen, Waltham, MA, USA) and Alexa Fluor 594-labeled goat anti-mouse IgG (A11032; Invitrogen) antibodies, respectively. Fluorescent images were acquired for six fields in a plate from 5 independent experiments and analyzed by using an Operetta CLS High-Content Imaging System (PerkinElmer, Waltham, MA, USA) for quantitative image analysis of maximum neurite length and average neurite length.

### 4.11. Statistical Analysis

The results were expressed as the means ± standard deviation (SD). Statistical analyses among multiple groups were performed by using analysis of variance (ANOVA), followed by the Tukey test as a post hoc test. *p* values of less than 0.05 were considered to indicate statistical significance.
